# A fever-based therapeutic window for bronchoscopy to prevent bronchiolitis obliterans in children with Mycoplasma pneumoniae pneumonia

**DOI:** 10.3389/fped.2026.1913026

**Published:** 2026-09-16

**Authors:** Mingming Cui, Lin Li, Cuiting Zhang, Weiyu Zhao, Ruiying Fan, Guiju Zhang, Qianqian Li

**Affiliations:** 1 Department of Pediatrics, The First Clinical College of Shandong University of Chinese Medicine, Jinan, China; 2 Department of Pediatrics, The Affiliated Hospital of Shandong University of Chinese Medicine, Jinan, China; 3 Department of Pediatrics, Haiyang Hospital of Chinese Medicine, Haiyang, China; 4 Department of Internal Medicine, People’s Hospital of Shizhong District, Jinan, Shandong, China

**Keywords:** bronchiolitis obliterans, fever-to-bronchoscopy time, flexible bronchoscopy, Mycoplasma pneumoniae pneumonia, pediatric pneumonia

## Abstract

**Background:**

Flexible bronchoscopy is frequently used in children with severe or refractory Mycoplasma pneumoniae pneumonia (MPP), but the optimal timing of intervention remains uncertain. This study examined the nonlinear association between time from fever onset to bronchoscopy and post-infectious bronchiolitis obliterans (BO), and explored whether a timing interval associated with the lowest observed risk could be identified.

**Methods:**

In this retrospective cohort study, 332 children with laboratory-confirmed MPP who underwent flexible bronchoscopy during the index hospitalization were included. Restricted cubic spline modeling was used to evaluate the dose-response association between bronchoscopy timing and BO. Propensity score matching compared children treated within the spline-defined optimal window with those undergoing delayed bronchoscopy. Secondary outcomes included bronchoscopic complexity, post-bronchoscopy length of stay, and defervescence within 48 h.

**Results:**

Post-infectious BO occurred in 46 children (13.9%). BO incidence was lowest among children undergoing bronchoscopy at 4–5 days after fever onset and highest among those treated at ≥8 days. Restricted cubic spline analysis demonstrated a significant non-linear association between bronchoscopy timing and BO, with the lowest estimated risk around days 4–5 and a progressive increase after day 7. In the matched cohort, BO occurred in 7.4% of children in the 4–5-day group versus 22.1% in the delayed group, corresponding to a matched odds ratio of 0.28. The 4–5-day group also had lower bronchoscopic complexity, shorter post-bronchoscopy hospitalization, and higher 48 h defervescence rates.

**Conclusions:**

Bronchoscopy timing in pediatric MPP was nonlinearly associated with post-infectious BO, with the lowest observed risk around days 4–5.

## Introduction

Mycoplasma pneumoniae pneumonia (MPP) remains an important cause of community-acquired pneumonia in school-aged children and adolescents, with periodic epidemic activity and substantial variation in clinical severity. Recent surveillance from China and Europe has shown renewed pediatric M. pneumoniae activity after the COVID-19 pandemic period, including increases in hospitalized and clinically severe cases (1, 2). This resurgence has occurred alongside persistent concern about macrolide-resistant M. pneumoniae, which has been reported in children in both Asian and non-Asian settings and may contribute to delayed clinical response in selected patients (3–5).

Although many children with MPP recover uneventfully, a clinically important subgroup develops severe or refractory disease characterized by persistent fever, progressive consolidation, pleural effusion, atelectasis, mucus plugging, plastic bronchitis, thrombotic complications, and post-infectious bronchiolitis obliterans (BO) (6, 7). These complications are thought to reflect a combination of pathogen-related epithelial injury, excessive host inflammatory response, mucus hypersecretion, impaired airway clearance, and activation of coagulation pathways. Several recent pediatric studies have identified inflammatory and coagulation biomarkers, including C-reactive protein (CRP), lactate dehydrogenase (LDH), and D-dimer, as useful markers of refractory or severe MPP (8, 9). However, these markers help identify risk; they do not clarify when airway-directed intervention is most likely to prevent irreversible sequelae.

Flexible bronchoscopy with bronchoalveolar lavage (BAL) is commonly used in severe or refractory MPP when lobar consolidation, atelectasis, suspected mucus plugging, or plastic bronchitis is present. Prior work suggests that early bronchoscopic intervention may reduce disease burden in selected children with severe MPP, but the optimal timing remains uncertain (10). In practice, bronchoscopy is often delayed until persistent fever or radiographic progression becomes obvious, whereas very early bronchoscopy may be performed before obstructive airway lesions are fully established. This creates a clinically relevant question: whether there is a therapeutic window in which bronchoscopy is neither too early nor too late.

Therefore, this study evaluated the association between time from fever onset to bronchoscopy and post-infectious BO in children with laboratory-confirmed MPP undergoing flexible bronchoscopy. We aimed to determine whether the relationship between bronchoscopy timing and BO was non-linear, to identify a clinically meaningful timing window, and to assess whether this window was also associated with bronchoscopic complexity, post-bronchoscopy hospital stay, and early defervescence.

## Methods

### Study design, setting, and ethics

This was a retrospective cohort study conducted at The Affiliated Hospital of Shandong University of Chinese Medicine from January 2022 to December 2025. The study protocol was reviewed and approved by the Institutional Review Board of The First Clinical College of Shandong University of Chinese Medicine. Written informed consent was obtained from every patient. All methods were performed in accordance with the relevant guidelines and regulations, and the reporting conforms to the STROBE statement.

### Patient identification and eligibility criteria

Children were eligible if they met all of the following criteria: age younger than 18 years at admission; clinical and radiological diagnosis of community-acquired pneumonia; laboratory evidence of M. pneumoniae infection, defined as a positive polymerase chain reaction assay from a respiratory specimen and/or a serum M. pneumoniae IgM antibody titer of at least 1:160 at any point during the illness; and receipt of the first flexible bronchoscopy during the index hospitalization.

The decision to perform bronchoscopy was made by the treating clinical team. Common indications included persistent high fever despite macrolide therapy, lobar or multilobar consolidation, atelectasis, suspected mucus plugging, or clinical suspicion of plastic bronchitis.

Patients were excluded if they had microbiologically confirmed respiratory co-infection, including positive tests for other respiratory viruses, bacteria, or atypical pathogens. Patients were also excluded if they had major underlying chronic pulmonary, cardiac, or immune-related conditions, including congenital heart disease, bronchopulmonary dysplasia, primary or secondary immunodeficiency, cystic fibrosis, primary ciliary dyskinesia, asthma, or interstitial lung disease.

### Exposure definition

The primary exposure was bronchoscopy timing, defined as the number of days from documented fever onset to the start of the first bronchoscopy procedure. Fever onset was defined as the first day on which body temperature reached at least 38.0°C.

Bronchoscopy timing was analyzed both as a continuous variable and as a categorical variable. For categorical analyses, patients were divided into four timing groups according to the hypothesized therapeutic window: hyper-early bronchoscopy, bronchoscopy timing ≤3 days; early bronchoscopy, bronchoscopy timing 4–5 days; intermediate bronchoscopy, bronchoscopy timing 6–7 days; and late bronchoscopy, bronchoscopy timing ≥8 days. The intermediate group was selected as the reference group.

### Covariates

Candidate covariates were selected *a priori* based on clinical relevance and potential association with both bronchoscopy timing and subsequent outcomes. Baseline demographic variables included age, sex, and weight at admission. Clinical severity variables included maximum body temperature, respiratory rate, and oxygen requirement before bronchoscopy. Laboratory variables included CRP, LDH, procalcitonin (PCT), the neutrophil-to-lymphocyte ratio (NLR), D-dimer, and macrolide-resistance status, which was categorized as macrolide-resistant, macrolide-sensitive, or unavailable.

Radiographic severity was assessed using chest imaging before bronchoscopy and categorized according to the presence of single-lobe vs. multilobe consolidation, defined as involvement of two or more lobes. Pleural effusion was recorded as present or absent. Pre-bronchoscopy treatment variables included cumulative corticosteroid dose and duration of macrolide therapy.

### Outcomes

The primary outcome was post-infectious BO. Diagnosis required both of the following: new persistent or recurrent respiratory symptoms, including cough, wheezing, or dyspnea, lasting for at least 6 weeks after the acute M. pneumoniae infection; high-resolution CT performed at least 3 months after discharge showing evidence of small-airway disease.

Secondary outcomes included intraoperative bronchoscopic complexity, length of post-bronchoscopy hospital stay (LOS), and defervescence within 48 h after bronchoscopy. Intraoperative bronchoscopic complexity was evaluated using the Intraoperative Bronchoscopic Complexity Score (IBCC), an ordinal three-level score derived from the bronchoscopy report: grade 1, mild disease, defined as patent airways with non-viscous secretions easily cleared by suction; grade 2, moderate disease, defined as airways filled with viscous secretions requiring repeated lavage; and grade 3, severe disease, defined as obstructive tenacious bronchial casts or plastic bronchitis requiring specialized retrieval with forceps, basket, or cryoprobe. Defervescence within 48 h was defined as sustained body temperature ≤37.3°C for at least 24 consecutive hours within 48 h after bronchoscopy.

### Statistical analysis

Data were analyzed using R software [4.5.3]. A two-tailed *P*-value < 0.05 was considered statistically significant for all tests.

#### Step 1: Baseline characterization and unadjusted comparisons

We summarized and compared the baseline characteristics of the four bronchoscopy timing groups. Continuous variables were presented as medians with interquartile ranges (IQR) and compared using the Kruskal–Wallis test. Categorical variables were presented as counts with percentages and compared using the chi-squared test or Fisher's exact test.

#### Step 2: Testing the non-linear hypothesis for the primary outcome

To identify a potential optimal time window, we explicitly modeled bronchoscopy timing as a continuous variable to predict the risk of developing BO. We used a multivariable logistic regression model in the full, unmatched cohort, adjusting for all pre-specified covariates. Within this model, bronchoscopy timing was fitted using restricted cubic splines (RCS) with three knots placed at the 10th, 50th, and 90th percentiles of its distribution. This method allows the data to reveal the shape of the association and to identify a statistically significant threshold. The results were visualized as a dose-response curve, estimating the adjusted odds ratio (OR) for BO against a continuous bronchoscopy timing.

#### Step 3: RCS-informed propensity score matching (PSM) analysis

The primary matched analysis compared children undergoing bronchoscopy within the RCS-defined optimal window (bronchoscopy timing 4–5 days) with those undergoing delayed bronchoscopy (bronchoscopy timing ≥8 days). Propensity scores were estimated using logistic regression representing the probability of undergoing bronchoscopy within the optimal window. The propensity model included age, sex, body weight, fever-to-admission interval, maximum temperature, respiratory rate, oxygen requirement, CRP, LDH, PCT, D-dimer, multilobe consolidation, atelectasis, pleural effusion, and macrolide-resistance status. Continuous variables were entered in continuous form, and macrolide-resistance status was modeled as a three-level categorical variable. Patients were matched 1:1 using nearest-neighbor matching with a caliper of 0.2 standard deviations of the logit of the propensity score. Covariate balance was evaluated before and after matching using absolute standardized mean differences (SMDs), with values <0.10 indicating satisfactory balance.

#### Step 4: Analysis of secondary outcomes

For the full cohort, we constructed separate multivariable models to assess the independent association between bronchoscopy timing groups determined by RCS and the other secondary outcomes. LOS was analyzed using multiple linear regression, and the achievement of 48 h defervescence was analyzed using binary logistic regression. All models were adjusted for the same set of covariates.

Several sensitivity analyses were performed. First, the analysis was repeated after excluding patients who received a cumulative corticosteroid dose above the 75th percentile before bronchoscopy. Second, a comparison was performed between the hyper-early group (bronchoscopy timing ≤3 days) and the optimal-window group (bronchoscopy timing 4–5 days). In addition, a threshold-based sensitivity analysis compared timely bronchoscopy, defined as bronchoscopy timing ≤7 days, with delayed bronchoscopy, defined as bronchoscopy timing ≥8 days.

## Results

### Study population

During the study period, 486 children were assessed for eligibility. After screening, 332 children were included in the analysis (Supplementary Figure S1). According to bronchoscopy timing, 58 children (17.5%) underwent hyper-early bronchoscopy within 3 days of fever onset, 92 (27.7%) underwent early bronchoscopy at 4–5 days, 103 (31.0%) underwent intermediate bronchoscopy at 6–7 days, and 79 (23.8%) underwent late bronchoscopy at 8 days or later. The median age of the overall cohort was 6.4 years, and 176 children (53.0%) were boys. Children in the late bronchoscopy timing group tended to be older and had slightly higher body weight, longer fever-to-admission interval, longer pre-bronchoscopy macrolide exposure, and greater cumulative corticosteroid exposure than those in the earlier bronchoscopy timing groups. Inflammatory burden differed across bronchoscopy timing groups, with higher CRP, LDH, PCT, NLR, and D-dimer levels observed particularly in the hyper-early and late bronchoscopy groups. Multilobe consolidation was present in 65.5%, 54.3%, 40.8%, and 64.6% of the ≤3-day, 4–5-day, 6–7-day, and ≥8-day groups, respectively (*P* = 0.003), while atelectasis occurred in 37.9%, 29.3%, 22.3%, and 39.2%, respectively (*P* = 0.056). Macrolide-resistance status did not differ significantly across groups (Table 1).

**Table 1:** Baseline characteristics and indication-related features according to bronchoscopy timing.

Characteristic	Overall *N* = 332	Hyper-early ≤3 d *n* = 58	Early 4–5 d *n* = 92	Intermediate 6–7 d *n* = 103	Late ≥8 d *n* = 79	*P* value
Age, years	6.4 (4.9–7.6)	5.6 (4.4–6.8)	6.2 (4.8–7.4)	6.3 (4.9–7.5)	7.1 (5.6–8.4)	0.021
Male sex	176 (53.0)	31 (53.4)	49 (53.3)	55 (53.4)	41 (51.9)	0.997
Weight, kg	23.6 (19.1–28.7)	21.2 (17.5–25.0)	23.2 (19.1–28.1)	24.1 (19.4–28.6)	25.8 (20.5–31.3)	0.044
Fever-to-admission time, days	3.8 (2.8–5.0)	2.0 (1.5–2.6)	3.3 (2.7–4.1)	4.2 (3.4–5.1)	5.4 (4.5–6.5)	<0.001
Maximum temperature, °C	39.3 (38.9–39.6)	39.5 (39.1–39.8)	39.2 (38.9–39.5)	39.2 (38.8–39.5)	39.4 (39.0–39.7)	0.018
Respiratory rate, breaths/min	29 (25–33)	31 (27–34)	29 (26–32)	28 (24–31)	30 (27–34)	0.006
Oxygen requirement before bronchoscopy	69 (20.8)	16 (27.6)	18 (19.6)	13 (12.6)	22 (27.8)	0.041
CRP, mg/L	64.8 (45.6–88.5)	69.5 (50.2–94.0)	60.8 (43.7–82.6)	56.2 (40.8–78.1)	78.4 (57.3–106.2)	0.004
LDH, U/L	428 (368–492)	415 (362–468)	432 (376–506)	392 (341–442)	472 (417–548)	<0.001
PCT, ng/mL	0.11 (0.07–0.18)	0.12 (0.08–0.20)	0.10 (0.06–0.16)	0.09 (0.06–0.14)	0.14 (0.09–0.23)	0.022
NLR	5.1 (3.8–7.1)	5.6 (3.9–7.7)	4.9 (3.7–6.8)	4.5 (3.2–6.3)	5.9 (4.2–7.6)	0.037
D-dimer, mg/L FEU	0.76 (0.54–1.05)	0.78 (0.56–1.04)	0.70 (0.51–0.98)	0.62 (0.44–0.89)	0.94 (0.70–1.22)	<0.001
Multilobe consolidation	181 (54.5)	38 (65.5)	50 (54.3)	42 (40.8)	51 (64.6)	0.003
Pleural effusion	85 (25.6)	19 (32.8)	22 (23.9)	18 (17.5)	26 (32.9)	0.058
Atelectasis	103 (31.0)	22 (37.9)	27 (29.3)	23 (22.3)	31 (39.2)	0.056
Macrolide resistance status						0.871
Resistant	235 (70.8)	42 (72.4)	65 (70.7)	69 (67.0)	59 (74.7)	
Sensitive	60 (18.1)	9 (15.5)	17 (18.5)	23 (22.3)	11 (13.9)	
Unavailable	37 (11.1)	7 (12.1)	10 (10.9)	11 (10.7)	9 (11.4)	
Macrolide therapy before bronchoscopy, days	4.8 (3.1–6.3)	2.0 (1.2–2.7)	4.0 (3.1–4.8)	5.0 (4.3–5.8)	7.1 (6.2–8.0)	<0.001
Pre-bronchoscopy corticosteroid use	167 (50.3)	25 (43.1)	42 (45.7)	51 (49.5)	49 (62.0)	0.095
Cumulative corticosteroid dose, mg/kg	2.9 (1.4–5.6)	1.2 (0.6–2.2)	2.2 (1.1–3.8)	3.3 (1.7–6.1)	5.4 (3.0–8.2)	<0.001

Values are median (IQR) or *n* (%). Bronchoscopy indications were not mutually exclusive. Because a single principal indication was not consistently recorded, objective pre-bronchoscopy indication-related features are presented. CRP, C-reactive protein; LDH, lactate dehydrogenase; PCT, procalcitonin; NLR, neutrophil-to-lymphocyte ratio; FEU, fibrinogen-equivalent units. *P* values were derived using Kruskal–Wallis tests for continuous variables and *χ*^2^ or Fisher’s exact tests for categorical variables.

### Non-linear association between bronchoscopy timing and post-infectious BO

Post-infectious BO occurred in 46 of 332 children, yielding an overall incidence of 13.9%. The crude incidence of BO differed across bronchoscopy timing groups, with the lowest rate observed in the early bronchoscopy group. BO occurred in 8 of 58 children (13.8%) in the hyper-early group, 6 of 92 children (6.5%) in the early group, 14 of 103 children (13.6%) in the intermediate group, and 18 of 79 children (22.8%) in the late group (*P* = 0.018). In the multivariable model, LDH, D-dimer, multilobe consolidation, and cumulative corticosteroid dose remained independently associated with BO. Compared with the intermediate group, late bronchoscopy remained associated with a higher adjusted odds of BO, although the confidence interval (CI) was wide and the categorical comparison was borderline (adjusted OR, 2.07; 95% CI, 0.90–4.78; *P* = 0.087). Early bronchoscopy at 4–5 days retained the lowest adjusted point estimate (adjusted OR, 0.41; 95% CI, 0.14–1.19; *P* = 0.102) (Table 2).

**Table 2:** Univariable and multivariable logistic regression analyses for post-infectious bronchiolitis obliterans.

Variable	Univariable		*P*	Multivariable		*P*
	OR	95% CI		OR	95% CI	
Bronchoscopy timing group						
Intermediate	Reference	—	—	Reference	—	—
Hyper-early	1.02	0.39–2.66	0.965	0.91	0.31–2.67	0.864
Early	0.44	0.16–1.20	0.109	0.41	0.14–1.19	0.102
Late	1.88	0.88–4.02	0.103	2.07	0.90–4.78	0.087
Age, per 1-year increase	1.12	1.01–1.28	0.044	1.08	0.93–1.25	0.307
Male sex	1.08	0.58–2.00	0.814	—	—	—
Weight, per 5-kg increase	1.18	0.77–1.44	0.196	—	—	—
Fever-to-admission time, per 1-day increase	1.28	0.80–1.49	0.222	—	—	—
Maximum temperature, per 0.5 °C increase	1.25	0.88–1.60	0.171	—	—	—
Respiratory rate, per 5 breaths/min increase	1.46	0.87–1.99	0.218	—	—	—
Oxygen requirement before bronchoscopy	2.36	0.81–4.59	0.312	—	—	—
CRP, per 10-mg/L increase	1.13	1.04–1.22	0.003	1.08	0.98–1.19	0.118
LDH, per 100-U/L increase	1.51	1.17–1.95	0.002	1.34	1.01–1.80	0.046
PCT, per 0.1-ng/mL increase	1.21	1.02–1.44	0.032	1.11	0.91–1.36	0.301
NLR, per 1-unit increase	1.14	0.83–1.26	0.109	—	—	—
D-dimer, per 0.5-mg/L FEU increase	1.43	1.16–1.77	0.001	1.29	1.01–1.65	0.043
Multilobe consolidation	3.02	1.51–6.04	0.002	2.21	1.03–4.75	0.042
Pleural effusion	2.14	1.11–4.13	0.023	1.59	0.76–3.32	0.219
Atelectasis	1.84	0.87–3.48	0.161	—	—	—
Macrolide resistance status						
Sensitive	Reference	—	—	—	—	—
Resistant	1.31	0.55–3.13	0.543	—	—	—
Unavailable	1.09	0.31–3.79	0.894	—	—	—
Macrolide therapy before bronchoscopy, per 1-day increase	1.22	0.88–1.38	0.101	—	—	—
Pre-bronchoscopy corticosteroid use	1.76	0.74–3.30	0.118	—	—	—
Cumulative corticosteroid dose, per 1-mg/kg increase	1.15	1.06–1.24	<0.001	1.1	1.00–1.21	0.049

BO, bronchiolitis obliterans; OR, odds ratio; CI, confidence interval; CRP, C-reactive protein; LDH, lactate dehydrogenase; PCT, procalcitonin; NLR, neutrophil-to-lymphocyte ratio; FEU, fibrinogen-equivalent units.

bronchoscopy timing was then modeled as a continuous variable using RCS in a multivariable logistic regression model adjusted for age, CRP, LDH, PCT, D-dimer, multilobe consolidation, pleural effusion, and pre-procedural cumulative corticosteroid dose. The spline model suggested a non-linear association between bronchoscopy timing and BO risk, with the lowest estimated risk observed when bronchoscopy was performed approximately 4–5 days after fever onset. The overall association between bronchoscopy timing and BO was statistically significant (overall *P* = 0.006), and the non-linear component was also significant (*P* for non-linearity = 0.031). Using bronchoscopy timing day 5 as the reference point, the adjusted OR for BO remained close to unity before day 6 but increased progressively thereafter. The adjusted OR was 1.20 at day 6, 1.55 at day 7, 2.23 at day 8, 3.04 at day 9, and 3.86 at day 10. The lower bound of the 95% CI crossed above 1.0 at approximately 7.6 days, suggesting that bronchoscopy performed after the late day-7 to early day-8 period was associated with a statistically significant increase in BO risk. In contrast, bronchoscopy performed within 3 days of fever onset was not associated with a lower adjusted BO risk than bronchoscopy performed at 4–5 days. The corresponding 95% CIs were 1.24–4.03 at day 8, 1.55–5.97 at day 9, 1.82–8.21 at day 10, and 2.21–15.80 at day 12 (Figure 1 and Table 3).

**Figure 1:**
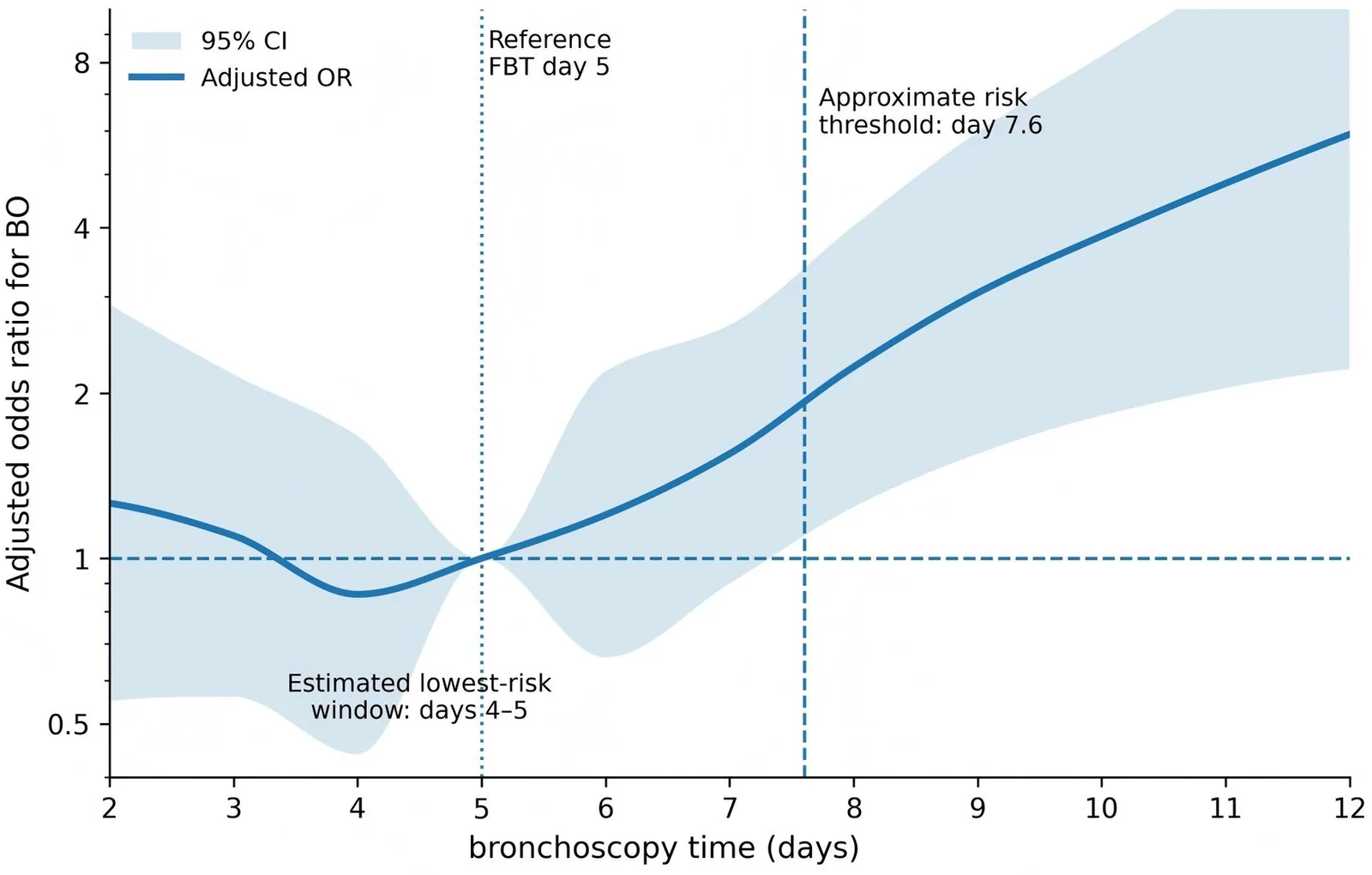
Nonlinear association between bronchoscopy time and post-infectious bronchiolitis obliterans (BO).

**Table 3:** Adjusted odds ratios for BO across continuous bronchoscopy timing values from the restricted cubic spline model.

Bronchoscopy timing, days	Adjusted OR for BO	95% CI
2	1.26	0.55–2.90
3	1.1	0.56–2.16
4	0.86	0.44–1.67
5	1	Reference
6	1.2	0.66–2.19
7	1.55	0.90–2.66
8	2.23	1.24–4.03
9	3.04	1.55–5.97
10	3.86	1.82–8.21
12	5.91	2.21–15.80

BO, bronchiolitis obliterans; OR, odds ratio; CI, confidence interval.

### RCS-informed PSM analysis

Propensity score matching generated 68 matched pairs. The mean absolute SMD decreased from 0.400 before matching to 0.046 after matching, and the maximum absolute SMD decreased from 1.530 to 0.083. All post-matching absolute SMDs were <0.10, indicating satisfactory covariate balance (Supplementary Figure S2).

In the matched cohort, post-infectious BO occurred less frequently in the optimal-window group than in the delayed group. BO developed in 5 of 68 children (7.4%) who underwent bronchoscopy at 4–5 days and 15 of 68 children (22.1%) who underwent bronchoscopy at ≥8 days. The absolute risk difference between the groups was −14.7 percentage points, and the matched OR was 0.28 (95% CI, 0.10–0.84; *P* = 0.023).

### Secondary outcomes in the full cohort

Bronchoscopy timing was regrouped into three clinically interpretable categories: hyper-early bronchoscopy (≤3 days; *n* = 58), optimal-window bronchoscopy (4–5 days; *n* = 92), and later bronchoscopy (≥6 days; *n* = 182). The 4–5-day group was used as the reference category because this interval corresponded to the lowest estimated risk in the restricted cubic spline analysis.

IBCC grade 3 disease was least frequent in the 4–5-day group and most frequent among children undergoing bronchoscopy at ≥6 days. Specifically, grade 3 complexity was observed in 10 of 58 children (17.2%) in the ≤3-day group, 8 of 92 children (8.7%) in the 4–5-day group, and 45 of 182 children (24.7%) in the ≥6-day group. Conversely, grade 1 findings were most common in the 4–5-day group, occurring in 39 children (42.4%), compared with 17 children (29.3%) in the ≤3-day group and 36 children (19.8%) in the ≥6-day group. Overall, the distribution of bronchoscopic complexity differed significantly across bronchoscopy timing groups (*P* < 0.001), supporting a U-shaped pattern in which both very early and later bronchoscopy were associated with greater procedural complexity than bronchoscopy performed during days 4–5. (Table 4)

**Table 4:** Secondary outcomes in the full cohort.

Outcome	Bronchoscopy timing ≤3 d *n* = 58	Bronchoscopy timing 4–5 d *n* = 92	Bronchoscopy timing ≥6 d *n* = 182	*P* value
IBCC grade				<0.001
Grade 1	17 (29.3)	39 (42.4)	36 (19.8)	
Grade 2	31 (53.4)	45 (48.9)	101 (55.5)	
Grade 3	10 (17.2)	8 (8.7)	45 (24.7)	
Post-bronchoscopy LOS, days	6.3 (4.8–8.0)	5.1 (4.0–6.8)	7.0 (5.4–9.2)	<0.001
Defervescence within 48 h	34 (58.6)	70 (76.1)	95 (52.2)	<0.001
Adjusted model summary				
Model	Comparison	Adjusted estimate	95% CI	*P* value
Linear regression for LOS	≤3 d vs. 4–5 d	*β* = 0.58 days	0.06–1.11	0.03
Linear regression for LOS	≥6 d vs. 4–5 d	*β* = 1.21 days	0.75–1.66	<0.001
Logistic regression for 48 h defervescence	≤3 d vs. 4–5 d	OR = 0.49	0.25–0.97	0.041
Logistic regression for 48 h defervescence	≥6 d vs. 4–5 d	OR = 0.36	0.20–0.65	<0.001

Values are *n* (%) or median (IQR). IBCC, intraoperative bronchoscopic complexity score; LOS, length of hospital stay. Adjusted models included age, CRP, LDH, PCT, D-dimer, multilobe consolidation, pleural effusion, and cumulative pre-bronchoscopy corticosteroid dose.

The median post-bronchoscopy LOS was shortest in the 4–5-day group, at 5.1 days (IQR, 4.0–6.8), compared with 6.3 days (IQR, 4.8–8.0) in the ≤3-day group and 7.0 days (IQR, 5.4–9.2) in the ≥6-day group. In the multivariable linear regression model adjusted for age, CRP, LDH, PCT, D-dimer, multilobe consolidation, pleural effusion, and cumulative pre-bronchoscopy corticosteroid dose, both non-optimal timing groups remained independently associated with longer post-bronchoscopy LOS. Compared with bronchoscopy at 4–5 days, bronchoscopy at ≤3 days was associated with an adjusted increase of 0.58 days in LOS (*β*, 0.58; 95% CI, 0.06–1.11; *P* = 0.030), whereas bronchoscopy at ≥6 days was associated with an adjusted increase of 1.21 days (*β*, 1.21; 95% CI, 0.75–1.66; *P* < 0.001) (Table 4).

Defervescence within 48 h after bronchoscopy was achieved most frequently in the 4–5-day group. Sustained defervescence occurred in 34 of 58 children (58.6%) in the ≤3-day group, 70 of 92 children (76.1%) in the 4–5-day group, and 95 of 182 children (52.2%) in the ≥6-day group (*P* < 0.001). After adjustment for the same pre-procedural covariates, both hyper-early and later bronchoscopy were associated with lower odds of 48 h defervescence compared with bronchoscopy at 4–5 days. The adjusted OR was 0.49 (95% CI, 0.25–0.97; *P* = 0.041) for the ≤3-day group and 0.36 (95% CI, 0.20–0.65; *P* < 0.001) for the ≥6-day group (Table 4).

### Sensitivity analyses

First, after excluding children who received a cumulative pre-bronchoscopy corticosteroid dose above the 75th percentile, 249 children remained in the analysis. The 75th percentile of cumulative corticosteroid exposure was 5.6 mg/kg. In this restricted cohort, BO occurred in 26 of 249 children (10.4%). The crude incidence of BO remained lowest among children who underwent bronchoscopy at 4–5 days. BO occurred in 7 of 54 children (13.0%) in the ≤3-day group, 5 of 80 children (6.3%) in the 4–5-day group, 8 of 75 children (10.7%) in the 6–7-day group, and 6 of 40 children (15.0%) in the ≥8-day group. In the adjusted model, bronchoscopy at 4–5 days retained the lowest risk estimate, whereas bronchoscopy at ≥8 days remained directionally associated with increased BO risk compared with 4–5 days (adjusted OR, 2.45; 95% CI, 0.68–8.76; *P* = 0.169). Repeating the RCS model in this restricted cohort yielded a similar curve shape, with the nadir remaining at approximately 4–5 days and the estimated odds of BO rising after day 7. The overall association between bronchoscopy timing and BO remained significant (overall *P* = 0.028), with borderline evidence of non-linearity (*P* for non-linearity = 0.049) (Figure 2).

**Figure 2:**
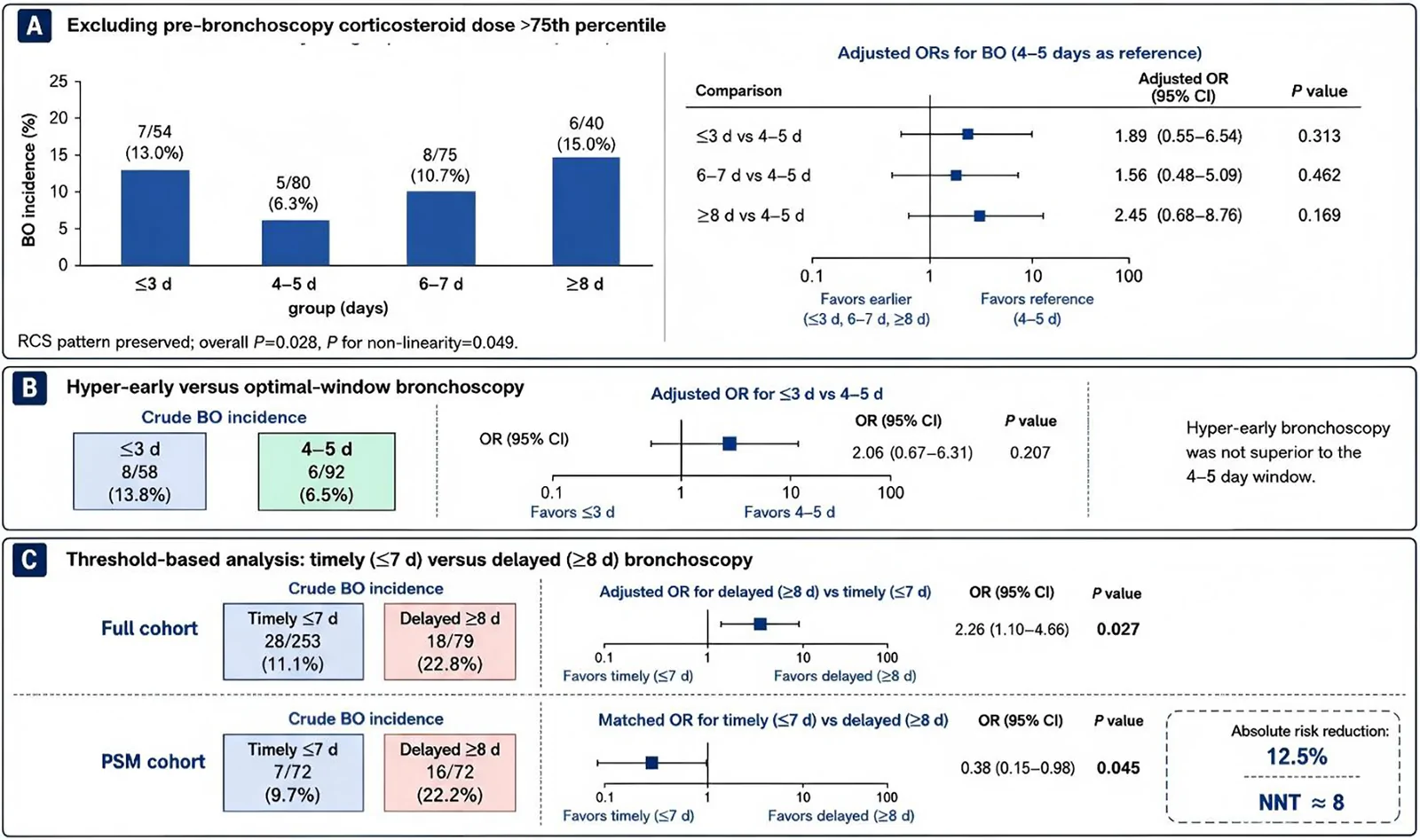
Sensitivity analyses of the association between bronchoscopy timing and post-infectious bronchiolitis obliterans (BO). **(A)** for excluding pre-bronchoscopy corticosteroid dose >75th percentile; **(B)** for hyper-early versus optimal-window bronchoscopy; **(C)** for Threshold-based analysis: timely (<7 d) versus delayed(≥8 d) bronchoscopy.

Second, BO occurred in 8 of 58 children (13.8%) in the ≤3-day group and 6 of 92 children (6.5%) in the 4–5-day group. In the multivariable logistic regression model, hyper-early bronchoscopy was not associated with a lower risk of BO compared with bronchoscopy at 4–5 days. Instead, the adjusted point estimate favored the 4–5-day group, although the difference did not reach statistical significance (adjusted OR for ≤3 days vs. 4–5 days, 2.06; 95% CI, 0.67–6.31; *P* = 0.207) (Figure 2).

Third in the full cohort, BO occurred in 28 of 253 children (11.1%) in the timely group and 18 of 79 children (22.8%) in the delayed group (*P* = 0.009). After adjustment for the same pre-procedural covariates, delayed bronchoscopy remained independently associated with higher odds of BO compared with timely bronchoscopy (adjusted OR, 2.26; 95% CI, 1.10–4.66; *P* = 0.027). In the corresponding PSM sensitivity analysis, 72 matched pairs were generated between the timely and delayed groups. After matching, all measured covariates achieved acceptable balance, with absolute SMDs <0.1. BO occurred in 7 of 72 children (9.7%) in the ≤7-day group and 16 of 72 children (22.2%) in the ≥8-day group. The absolute risk difference was −12.5 percentage points, and the matched OR was 0.38 (95% CI, 0.15–0.98; *P* = 0.045) (Figure 2).

## Discussion

This study identified a nonlinear relationship between bronchoscopy timing and post-infectious BO in children with MPP selected for bronchoscopy. Rather than supporting a simple “earlier is always better” interpretation, the findings suggest a discrete lowest-risk interval around days 4–5 after fever onset, followed by increasing risk from approximately late day 7 to early day 8. This result extends previous studies of severe MPP, which generally evaluated timing using broad categories such as early versus delayed treatment, time from admission, or radiographic response, rather than as a continuous fever-based exposure anchored to illness onset (10–12). The analysis therefore provides clinically useful granularity: the relevant question may concern not merely whether bronchoscopy is “early,” but whether it is performed while airway obstruction is emerging and potentially reversible.

The observed timing pattern is biologically plausible. Severe and refractory MPP is increasingly recognized as both an infectious pneumonia and a dynamic airway disease. Studies of plastic bronchitis and refractory MPP have linked airway obstruction to inflammatory burden, secretion viscosity, mucus plug formation, atelectasis, pleural involvement, and bronchoscopically visible airway lesions (13, 14). Evidence from BAL fluid further supports prominent local inflammation, altered cytology, increased pathogen burden, oxidative stress, mucin-related airway hypersecretion, and proteomic changes in severe disease (15–19). In this context, bronchoscopy performed too early may precede the development of obstructive secretions or casts as the dominant therapeutic target, whereas a late procedure may follow sustained mucosal injury, small-airway inflammation, and organization of luminal material. The observed 4–5-day window may therefore mark an interval during which bronchoscopic clearance could interrupt evolving airway obstruction before fixed bronchiolar damage develops.

Focusing on post-infectious BO is clinically important. BO after severe MPP is a disabling sequela, and recent pediatric studies have emphasized the need to identify potentially preventable antecedents early in the acute illness (20). Longitudinal pulmonary function studies in pediatric post-infectious BO have shown persistent obstructive impairment in many children, even after prolonged follow-up (21). Broader pediatric cohorts have likewise identified features of severe lower respiratory infection and delayed recognition as relevant to BO development (22, 23). The present study extends this literature by shifting the question from “who is at risk” to “when bronchoscopy is associated with lower BO risk.” This distinction is clinically relevant because reducing BO risk may depend not only on recognizing severe disease but also on acting before the inflammatory-obstructive process becomes structurally established.

Importantly, the observed association cannot be separated completely from disease severity. Children undergoing later bronchoscopy are often older, have a longer pre-hospital fever duration, receive more pre-procedural treatment, and show a greater inflammatory or radiographic burden. These factors have been linked to refractory or severe MPP in several prediction studies, particularly CRP, LDH, D-dimer, and dense pulmonary consolidation (6–9). Adjustment for key pre-procedural covariates and the matched comparison reduced measured imbalance. Nevertheless, residual confounding remains possible, especially because corticosteroid exposure may represent both treatment and clinician-perceived disease severity. The sensitivity analysis excluding high corticosteroid exposure preserved the overall curve shape but could not fully distinguish confounding by indication from disease biology.

Macrolide resistance provides additional clinical context. Recent surveillance and clinical studies have shown substantial geographic variation in macrolide-resistant M. pneumoniae, with high rates in parts of China and variable clinical effects across settings (3–5). In the present cohort, resistance status did not differ significantly across bronchoscopy timing groups, making it unlikely that macrolide resistance alone explained the timing association. Persistent fever after macrolide therapy commonly triggers both antimicrobial escalation and consideration of bronchoscopy. The findings suggest that airway disease progression warrants separate attention even in the setting of macrolide resistance. Persistent fever should therefore prompt reassessment not only of antimicrobial adequacy but also of mucus plugging, atelectasis, lobar consolidation, and evolving plastic bronchitis.

Secondary analyses likewise showed that the 4–5-day group had a more favorable bronchoscopic complexity profile, shorter post-bronchoscopy hospitalization, and earlier defervescence than the other timing groups. However, these patterns may partly reflect disease severity at the time of bronchoscopy because the IBCC may capture severity rather than an effect of procedure timing itself. Comparable findings have been reported for bronchoscopic airway abnormalities and CT severity in refractory MPP (13). BAL studies have also identified inflammatory, oxidative, and mucin-related signals in the lower airway compartment that may contribute to persistent fever and impaired ventilation (15–19). Together, these observations provide a potential biological explanation for the associations with shorter hospitalization and earlier defervescence, but they do not establish that bronchoscopy timing caused these outcomes.

From a clinical perspective, the findings support a more nuanced approach than indiscriminate early bronchoscopy. Hyper-early bronchoscopy was not superior to the procedure performed on days 4–5, suggesting that intervention before airway lesions are established may add procedural burden without reducing long-term risk. Conversely, bronchoscopy on day 8 or later may coincide with prolonged airway obstruction and inflammatory injury. Children with MPP who have persistent high fever, lobar or multilobar consolidation, atelectasis, an oxygen requirement, elevated LDH or D-dimer, or suspected mucus plugging could therefore be reassessed proactively around days 4–5 after fever onset rather than after a full week of unsuccessful therapy (6–10). This finding does not imply that all children with MPP require bronchoscopy; the identified window should be used only to prioritize assessment among high-risk children who already meet clinical indications for airway evaluation.

Careful endpoint selection is also important in pediatric MPP research. Previous studies have often focused on fever duration, length of stay, radiographic resolution, or short-term treatment response. Although clinically relevant, these outcomes may not capture the long-term burden of small-airway injury. Using post-infectious BO as the primary outcome centers a sequela that is directly meaningful to children and families. However, BO diagnosis relied on follow-up symptoms and CT findings, and longitudinal pulmonary function trajectories were unavailable (24). Future studies should incorporate standardized spirometry or oscillometry, predefined imaging follow-up intervals, symptom scores, and quality-of-life measures to determine whether the observed timing window is associated with better long-term functional recovery.

Several limitations should be considered. First, this retrospective, single-center study included only children who underwent bronchoscopy, limiting generalizability. Only 46 patients developed BO; consequently, the multivariable models may have been overfitted and the effect estimates unstable. Second, because the decision to perform bronchoscopy and its timing were determined by treating clinicians rather than predefined criteria, timing differences may have reflected not only baseline disease severity but also institutional practice, physician preference, healthcare accessibility, and referral patterns. Propensity score matching reduced measured clinical differences but could not account for unmeasured clinical or system-level factors. Residual confounding may therefore have exaggerated the apparent benefit associated with bronchoscopy at 4–5 days and may limit the generalizability of the findings. Third, fever onset may have been subject to documentation or recall error. Fourth, RCS confidence bands widened at longer bronchoscopy timing values, indicating greater uncertainty in the right tail of the distribution. The magnitude and precise shape of the association at very late bronchoscopy timings should therefore be interpreted cautiously. Finally, the proposed timing window requires external validation in prospective, multicenter studies before it can inform procedural recommendations.

In conclusion, bronchoscopy timing showed a nonlinear association with post-infectious BO among children with MPP who underwent bronchoscopy, with the lowest observed risk around days 4–5. Given the retrospective observational design and potential residual confounding, no causal or preventive effect can be inferred. Prospective, multicenter studies are required before this timing interval can inform procedural recommendations.

## Data Availability

The original contributions presented in the study are included in the article/Supplementary Material, further inquiries can be directed to the corresponding authors.
